# Sensitive periods of child development: a study on the construction and validation of the BASENP mobile application

**DOI:** 10.1590/0034-7167-2024-0475

**Published:** 2025-12-08

**Authors:** Carolaine da Silva Souza, Paulo Roberto Gomes Abreu, Edmara Chaves Costa, Hévila Ferreira Gomes Medeiros Braga, Francisco Arnoldo Nunes de Miranda, Antônio Carlos da Silva Barros, Flávia Paula Magalhães Monteiro

**Affiliations:** I Universidade Federal do Rio Grande do Norte. Natal, Rio Grande do Norte, Brazil; II Universidade da Integração Internacional da Lusofonia Afro-Brazileira. Redenção, Ceará, Brazil

**Keywords:** Child Development, Methodological Study, Information Technology, Mobile Applications, Nursing, Desarrollo Infantil, Estudio Metodológico, Tecnología de la Información, Aplicaciones Móviles, Enfermaría.

## Abstract

**Objectives:**

to describe the construction and validation of a mobile application on sensitive periods of child development.

**Methods:**

methodological research focused on the technological development and validation of software, in the form of a mobile application, conducted from March to October 2023 in two stages: 1) construction of the BASENP mobile application and 2) content and appearance validation by experts.

**Results:**

content experts evaluated the material, resulting in a total Content Validity Index of 0.89. Experts in the field of information and communication technology obtained an Agreement Index of 0.86%. The binomial test was not significant for the majority of items in both groups.

**Conclusions:**

the application was developed and validated with methodological rigor and may contribute to improving knowledge and care provided to infants.

## RESUMEN

**Objetivos:**

describir la construcción y validación de una aplicación móvil sobre los períodos sensibles del desarrollo infantil.

**Métodos:**

investigación metodológica de producción tecnológica y validación de un *software*, en formato de aplicación móvil, llevada a cabo entre marzo y octubre de 2023, realizada en dos etapas: 1) Construcción de la aplicación móvil BASENP y 2) Validación del contenido y apariencia por parte de especialistas.

**Resultados:**

los especialistas en contenido permitieron la evaluación del material con un Índice de Validez de Contenido total de 0,89, mientras que los especialistas en tecnología de la información y comunicación obtuvieron un Índice de Concordancia de 0,86%. La prueba binomial no mostró significación estadística para la mayoría de los ítems en ambos grupos.

**Conclusiones:**

la aplicación fue construida y validada con rigor metodológico y puede contribuir a la mejora del conocimiento y de la atención brindada al lactante.

## INTRODUCTION

Child development is understood as a complex, continuous, dynamic, and progressive process that involves a set of initially simple changes, progressively evolving toward the acquisition of more advanced skills. As it is not a linear or uniform process, it goes through phases of disruption, mutation, and adaptation resulting from the relational dynamics of various physiological factors in the child’s life^(1-3)^.

Thus, upon reaching a new skill (milestone) in development, the child may undergo a process of acceleration, evidenced by behavioral disorganization, which manifests as disruptions in development and is subsequently followed by periods of reorganization. In general, these are accompanied by parental anxiety and crisis^(4)^.

In this context, on one hand, these skills may be popularly referred to by mothers and caregivers as “developmental leaps”, which are sensitive periods during which the child acquires new cognitive, motor, sensory, social, and language abilities, such as learning new words, writing, practicing a sport, among others. In reality, these are physiological changes that indicate the child is developing in a healthy way^(5)^.

On the other hand, the term “sensitive periods” in child development remains obscure and unfamiliar to many mothers and caregivers. Therefore, it is necessary to promote health education among parents and caregivers to disseminate and clarify this concept, emphasizing that these terms refer to the new skills the baby is acquiring at each developmental stage^(6)^.

Accordingly, parents and caregivers need to understand all dimensions of child development, which follow a defined and predictable sequence-i.e., children crawl before creeping, creep before standing, and stand before walking^(7,8)^.

As the child goes through these physiological stages, different behaviors may emerge, such as abrupt changes in sleep patterns, more frequent crying, increased irritability without an apparent cause, among others. In general, these behaviors reflect a need for attachment, attention, and affection^(9)^. In this sense, parents often feel confused and distressed and mistakenly fail to recognize which developmental stage their baby is experiencing, perceiving the process as “pathological”^(10)^.

As a result, parents frequently turn to informal sources of information, such as social media and personal blog websites, encountering unreliable content that can generate more fear and doubt rather than providing reassurance through accurate and scientific information^(5)^.

Moreover, there is still a lack of scientific evidence on sensitive periods that is accessible to this audience, with most of the content being shared informally among groups of mothers who have experienced similar challenges. Additionally, the topic is rarely addressed during consultations with healthcare professionals or in health education activities within child healthcare services^(6)^.

Therefore, the impact of technology on children’s health is an increasingly relevant topic, especially in an era where mobile devices dominate daily life. In summary, the use of these tools offers significant support and assistance to parents and caregivers, as the use of technological resources in the teaching and learning process is becoming increasingly necessary^(11)^.

Thus, this study is justified by the need to foster knowledge and develop a technology-such as a mobile application for parents and caregivers-focused on the sensitive periods of child development, considering the challenges encountered in infant care, as this is still an incipient topic in family discussions.

## OBJECTIVES

To describe the construction and validation of a mobile application on sensitive periods of child development.

## METHODS

### Ethical aspects

The study was conducted in accordance with national and international ethical guidelines and was approved by the Research Ethics Committee of the *Universidade da Integração Internacional da Lusofonia Afro-Brasileira* (UNILAB), with the approval document attached to this submission. The study followed the guidelines of Resolution No. 466/12. All participants signed the Informed Consent Form (ICF) prior to their inclusion in the study.

### Design, study setting, and period

This was a methodological study focused on the technological development and validation of software in the form of a mobile application^(12)^, conducted in two stages: (1) the construction of the BASENP mobile application, which took place between March and October 2023; and (2) the validation of content and appearance by experts, conducted online between September and October 2023. The Equator TRIPOD tool was used to guide the methodology, as it provides guidance for developing, validating, or updating multivariable prediction models^(13)^.

### Population or sample; inclusion and exclusion criteria

Once constructed, the mobile application was validated by a committee composed of healthcare professionals-specifically nurses with at least one year of clinical experience and theoretical knowledge in child development, a thesis or dissertation in the field, and publications on the topic-as well as information technology specialists with expertise in software development, evaluation, and/or implementation, in accordance with Fehring’s criteria^(14)^.

The selection of experts for validation followed the recommendations of NBR ISO/IEC 14598-6, which establishes a minimum of eight members for each group of evaluators to ensure the representativeness of the user category for software systems^(15)^.

### Study Protocol

This study followed the five stages proposed by Pressman and Maxim: communication, planning, modeling, construction, and delivery^(12)^. In the first stage, a search was conducted in virtual application stores-Apple Store and Play Store-to verify the existence of apps with similar themes. However, only one was found, which was paid, in a different language, and developed solely for commercial purposes.

It is noteworthy that, before confirming the name of the application, a search was also conducted on these same platforms to check for any existing patents or children’s materials with the same name.

Subsequently, a scoping review was developed to form the content of the technology, as registered on the OSF platform, under the protocol: https://doi.org/10.17605/OSF.IO/3JTDZ. Based on this review, the app’s content was defined to include: concepts related to sensitive periods, the number of periods, their timing, the domains of child development, developmental milestones, infant reactions at each stage, guidance for parents on how to handle these sensitive periods, and the importance of family involvement in this process.

In addition, a preliminary survey was conducted with parents and caregivers to identify their main needs related to the topic, contributing to the conceptual structure of the technology. These results are described in a broader study, derived from a master’s dissertation^(16)^.

The application was developed in partnership with the Computer Engineering program at the UNILAB, with support from a faculty member of that department and a volunteer student. Using the Figma platform, an online tool for creating prototype interfaces, it was necessary to build a model or prototype of the desired product, including the functions and features to be performed by the software.

Additionally, the incremental model was adopted as the development process, as it allows for the rapid delivery of a functional set of features to users, which can then be refined and expanded in subsequent versions^(17)^.

For the validation stage of the technology, expert recruitment was carried out through the Lattes Platform. Initial participants were identified using the keywords “child health”, “development”, “child development”, and “information and communication technology”, which served as the initial “seeds” of the study. In addition, the snowball sampling technique was used, in which participants from this phase referred other professionals with similar profiles to contribute to the evaluation of the material^(18)^.

The selected experts received an invitation letter-50 letters for each group of experts, totaling 100 invitations-along with the ICF and study instructions, sent via email. After the signed ICF was returned, the evaluation instrument and a link to access the application were made available. A 30-day period was granted for the return of the evaluated material, in line with the study’s objectives.

The validation of the application by the experts was divided into three parts: (1) collection of the expert’s profile (current occupation, area of expertise, academic degree, years of professional experience, and type of experience); (2) content evaluation using an adapted instrument employed in other mobile application validation studies^(19,20)^, composed of 18 items divided into the following domains: objectives, structure, presentation, and relevance; and (3) evaluation of the application regarding functionality, reliability, usability, efficiency, maintainability, and portability^(14)^.

Finally, each item in the evaluation instrument was rated on a five-point Likert scale, according to the experts’ assessments, ranging from “not at all appropriate” to “completely appropriate”^(21)^.

### Data Analysis and Statistics

The database was organized using Microsoft Excel, and data analysis was performed with the Statistical Package for the Social Sciences (SPSS), version 28.0. The Content Validity Index (CVI) was calculated to estimate the level of agreement among the items rated by the experts, with items considered valid if they achieved a score equal to or greater than 0.80 (19). In addition, the exact binomial distribution test-appropriate for small samples-was applied, with statistical significance set at p > 0.05 and a reference agreement threshold of 0.75 to estimate the statistical reliability of the CVI^(22)^.

## RESULTS

According to the process of construction and validation of the mobile application, the development of the content/conceptual structure of the software was based on 30 scientific studies and other materials, such as textbooks and websites, and followed the steps outlined below:

### Communication

The structure of the information was defined in terms of the type of language to be used, and it was established that the app would be aimed at parents and caregivers of infants. Additionally, the required features of the proposed application were defined as follows: a registration interface; fields for recording additional information; content about sensitive periods; supplementary materials; and terms and privacy policies.

### Planning

The planning focused on the layout structure, including colors and images, following a script based on the previously prepared content. The educational material was based on the scoping review described in the first stage of this study.

### Modeling

At this stage, the mobile app was prototyped, including the selection of the interfaces to be featured in the application. This required the assistance of a professional web designer to create the models previously defined during the planning stage, as well as the illustrations.

In total, 17 interfaces were included in the initial version of the prototype, with neutral pink and baby pink color palettes, and icons representing a baby, user, bell, settings, logs, and calendar. The illustrations used in the app were selected to promote diversity and social inclusion among its users.

### Construction

In this stage, the authors involved in the development of the technology used the SCRUM methodology, an agile approach commonly applied to more complex projects, involving the definition of basic concepts. The following key activities were carried out: creation of simple and intuitive interfaces; development for multiple ecosystems; use of a modern tool; and the selection of the Flutter framework for development^(23)^.

In the initial interface, users find a menu containing all relevant information, designed to highlight the images from the home screen and improve user understanding. Next, the section on sensitive periods is presented, containing all pertinent information on the topic. Any questions that parents/caregivers may have can be addressed by navigating through this interface and selecting the appropriate option.

Furthermore, in the interface titled Domains of Child Development, each domain is described: physical, cognitive, socio-emotional, and psychosocial. The following interface displays the developmental milestones from birth to two years of age. This screen provides guidance for parents on how to support their child when responding to a sensitive period. Finally, the app includes an interface explaining the importance of caregivers in the developmental process of their children.

Moving on, there is an interface where parents can record information of their choice regarding behaviors the baby is exhibiting or any questions that arise. This section includes a user profile, where users can register and input their information. It also includes the terms of use and a privacy policy to ensure confidentiality.

As for the color palette, green, sarje, and a neutral tone were chosen so the app could be used for all children, regardless of gender. The body text of the script was designed to present the information in an engaging manner, accompanied by widely relatable images of fathers, mothers, and grandparents, as well as representations of various child age groups, races, cultural backgrounds, social inclusion, and skin tones.

Subsequently, the authors themselves selected the name BASENP APP to reflect the content represented. The acronym “BA” stands for babys (baby), “SEN” for sensitive (sensíveis), and “P” for periods (períodos), resulting in “baby’s sensitive periods” (Babys sensitive periods).

### Delivery

**Figure 1:**
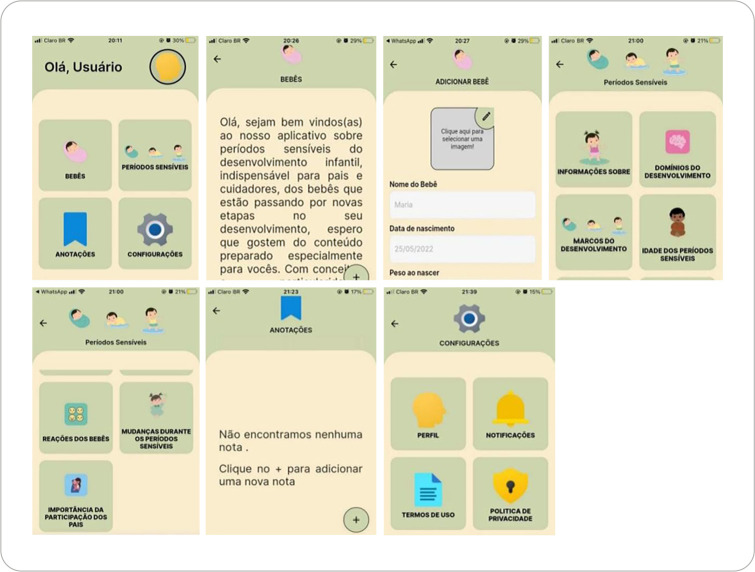
BASENP Mobile Application

In the delivery stage, the application was configured as a website (http://periodossensiveis.infinityfreeapp.com/#/config/), and it can be accessed on any type of device, including desktop computers and mobile phones, as shown in Figure 1.

Caption: Main Screen (*Olá, Usuário*)

*Bebês* → Babies

*Períodos Sensíveis* → Sensitive Periods

*Anotações* → Notes

*Configurações* → Settings

Welcome Message Screen (*Bebês*)

*Olá, sejam bem-vindos(as) ao nosso aplicativo sobre períodos sensíveis do desenvolvimento infantil, indispensável para pais e cuidadores, dos bebês que estão passando por novas etapas no seu desenvolvimento, espero que gostem do conteúdo preparado especialmente para vocês. Com conceito e particularidade de cada criança...*

*→ Hello, welcome to our application about sensitive periods of child development, an essential tool for parents and caregivers of babies who are going through new developmental stages. We hope you enjoy the content specially prepared for you, considering the concept and uniqueness of each child...*

Add Baby Screen (*Adicionar Bebê*)

*Clique aqui para selecionar uma imagem*! → Click here to select an image!

*Nome do Bebê* → Baby’s Name

*Data de nascimento* → Date of Birth

Sensitive Periods Menu (*Períodos Sensíveis*)

Informações Sobre → *About*

*Domínios do Desenvolvimento* → Developmental Domains

*Marcos do Desenvolvimento* → Developmental Milestones

*Idade dos Períodos Sensíveis* → Sensitive Period Ages

Sensitive Periods Submenu

*Reações dos Bebês* → Baby Reactions

*Mudanças Durante os Períodos Sensíveis* → Changes During Sensitive Periods

*Importância da Participação dos Pais* → Importance of Parental Involvement

Notes Screen (*Anotações*)

*Não encontramos nenhuma nota*. → We didn’t find any notes.

*Clique no + para adicionar uma nova nota* → Click the + to add a new note

Settings Menu (*Configurações*)

*Perfil* → Profile

*Notificações* → Notifications

*Termos de Uso* → Terms of Use

*Política de Privacidade* → Privacy Policy

### Content and appearance validation of the mobile application by experts

A total of 100 experts were invited; however, only 17 agreed to participate in the evaluation of the mobile application-nine nurses with expertise in child health and development, and eight professionals in the field of computing-reaching the minimum number defined for each group.

Among the nurses-eight of whom were female and one male-ages ranged from 24 to 58 years, with a mean age of 36.11 years. Regarding the year of degree completion, it ranged from 2007 to 2022. As for academic qualifications in the health group, four held master’s degrees, three held doctorates, and two had postdoctoral training.

It is also worth noting that among the health professionals, five were faculty members, three of whom were involved in both teaching and research. Table 1 below presents the experts’ agreement assessment on the content of the mobile application.

**Table 1:** Validation of item characteristics, Content Validity Index by content experts, Redenção, Ceará, Brazil, 2023

Item	No. of Agreements	CVI^*^	*p* value^**^
**OBJECTIVES**			
1. The objectives address the proposed topic	9	1.00	0.075
2. The mobile application is appropriate for the learning process about sensitive periods of development	9	1.00	0.075
3. The application clarifies doubts about the proposed topic	8	0.89	0.300
4. The application encourages reflection on the topic	9	1.00	0.075
5. The application contributes to parents’ and/or caregivers’ knowledge	9	1.00	0.075
**STRUCTURE / PRESENTATION**			
6. The language is appropriate for the target audience	6	0.67	0.010
7. The language is appropriate for the material covered	8	0.89	0.300
8. The language is interactive, allowing active engagement in the teaching-learning process	7	0.78	0.601
9. The application contains accurate information	8	0.89	0.300
10. The application contains objective information	8	0.89	0.300
11. The application contains clear information	8	0.89	0.300
12. The application contains necessary information	9	1.00	0.075
13. The application presents a logical sequence of ideas	9	1.00	0.075
14. The topic is up to date	9	1.00	0.075
15. The amount of text is appropriate	7	0.78	0.601
**RELEVANCE**			
16. The application encourages understanding of the topic	9	1.00	0.075
17. The application contributes to knowledge in the field	9	1.00	0.075
18. The application sparks interest in the topic	9	1.00	0.075
**PART III - FUNCTIONALITY**			
19. The application is easy to use	8	0.89	0.300
20. The application performs its functions accurately	8	0.89	0.300
21. The application presents the main features necessary for the teaching-learning process on the topic	8	0.89	0.300
**RELIABILITY**			
22. The application responds appropriately when errors occur	8	0.89	0.300
23. The application alerts the user when invalid data is entered	8	0.89	0.300
**USABILITY**			
24. It is easy to learn how to use the application	8	0.89	0.300
25. The application offers help in a clear way	6	0.67	0.010
**EFFICIENCY**			
26. The application’s execution time is appropriate	9	1.00	0.075
27. The resources provided in the application are adequate	7	0.78	0.601
**TOTAL CVI**		**0.89**	

* Content Validity Index;

** Binomial test.

Based on the above, it is possible to observe that items 6 (CVI = 0.67), 8 (CVI = 0.78), 15 (CVI = 0.78), and 25 (CVI = 0.67) did not reach consensus among the experts regarding “appropriate language for the target audience”, “interactive language allowing active involvement in the teaching-learning process”, “adequate amount of text”, and whether the app “offers clear help”, respectively. These items did not achieve a score above 0.80, as previously established in this study. In the binomial test, significant disagreement was observed in item 6 (p = 0.010).

Thus, it can be considered that the remaining items were deemed adequate according to the adopted index, as they achieved an average total CVI score of 0.89, which is considered significant for content and appearance validation.

In this evaluation, health experts were given the opportunity to provide suggestions, if they deemed necessary, to improve the mobile application. Table 2 below presents the appearance validation by technical experts.

**Table 2:** Validation of item characteristics, Content Validity Index by appearance experts, Redenção, Ceará, Brazil, 2023

Item	No. of Agreements	CVI^*^	*p* value^**^
**FUNCTIONALITY**			
1. The application performs its functions accurately	8	1.00	0.100
2. The application performs as intended	7	0.88	0.267
3. The application provides secure access through passwords	3	0.38	0.023
**RELIABILITY**			
4. The application responds appropriately when failures occur	7	0.88	0.267
5. The application informs the user when invalid data is entered	6	0.75	0.311
**USABILITY**			
6. It is easy to understand the concept of sensitive periods in child development and the use of the application	8	1.00	0.100
7. It is easy to learn how to use the application	8	1.00	0.100
8. The application offers help clearly	7	0.88	0.267
9. The application is easy to operate and control	8	1.00	0.100
**EFFICIENCY**			
10. The application’s execution time is appropriate	7	0.88	0.267
11. The resources provided are adequate	8	1.00	0.100
**MAINTAINABILITY**			
12. It is easy to identify a fault when it occurs	7	0.88	0.267
13. It is easy to modify and adjust the application when necessary	8	1.00	0.100
14. It is easy to test when changes are made to the application	8	1.00	0.100
**PORTABILITY**			
15. It is easy to adapt the application to other environments	7	0.88	0.267
16. It is easy to install the application on other devices	7	0.88	0.267
**TOTAL CVI**		**0.86**	

* Content Validity Index;

** Binomial test.

From this, it is possible to observe that items 3 (CVI = 0.38) and 5 (CVI = 0.75) did not reach consensus among the experts. In the binomial test, significant disagreement was observed for item 3 (p = 0.027).

Thus, it can be considered that the remaining items evaluated were deemed adequate according to the adopted index, as they achieved an average total CVI score of 0.86, which is considered significant for the technical validation of the application.

As for suggestions for improving the app made by the experts, the following were highlighted: spelling review; association of images with the child’s age group; inclusion of links, supplementary materials, videos, child avatars, and the ability to change the date of entries. These were the main suggestions accepted by the authors, and the application was updated accordingly based on the experts’ contributions.

## DISCUSSION

The mobile application is a computer program developed for use on mobile devices such as smartphones, tablets, computers, among others, and includes features designed specifically for educational contexts. Accordingly, the BASENP app was developed with the aim of providing health education to users, in an effort to fill a gap that still exists in the literature^(24)^.

Regarding the development of the application prototype, a layout was designed to capture users’ attention and promote interactivity, including videos from the Ministry of Health and images that reflect family realities, as well as the inclusion of vulnerable populations, with images of Black children, children with disabilities, children with albinism, among others. Studies that developed applications focused on child health emphasize the importance of supplementary materials to make the tool more dynamic^(25,26)^.

One study indicated that focusing on the app’s design and content facilitates accessibility and smooth navigation through screens, considering that the prototype is intended for parents and/or caregivers and must have an interactive and user-friendly interface-especially given that many parents are not accustomed to using mobile applications^(27)^.

Thus, the content included in the application was developed through a scoping review to identify the strengths and weaknesses related to the topic. It covers the context of sensitive periods as well as the reactions of each baby. This same strategy was used in another study to develop a mobile app for evaluating the feet of people with diabetes mellitus-one of the essential steps in building any technology intended to guide application development^(28)^.

In this context, family knowledge about child development-particularly regarding behaviors children exhibit throughout their developmental stages-is of fundamental importance, as it influences parental beliefs and practices and, consequently, the child’s development^(25)^.

Furthermore, it is emphasized that the construction and validation of technologies are essential and complex processes that require pedagogical strategies and appropriate methods. If validation is not carried out, the material becomes inadequate and fails to fulfill its intended purpose of providing educational information^(29)^.

Some authors point out that the functional adequacy and reliability of an application are directly linked to the technology’s ability to provide features that meet the population’s needs. In this regard, it is essential to evaluate quality to determine whether the technology offers appropriate functionality in one or more of its components^(24)^.

The BASENP app was considered valid, aiming to contribute educationally to parents and caregivers in monitoring the development of their children, as it provides information to address any questions that may arise. In this context, the importance of this technology is underscored in terms of support, knowledge dissemination, and the spread of information to the target audience^(22)^.

The use of mobile applications holds strong potential in educational activities, thus contributing to the teaching-learning process and further encouraging interest in research and the reading of bibliographic information^(24)^. The development of this application addresses the justification and the need to promote knowledge and introduce a technology-such as a mobile app-for parents and caregivers of children regarding the topic in question.

Regarding the topic addressed in this study, the literature reveals a gap in the development of educational materials, as well as in the availability of information on the subject, specifically concerning the care and monitoring of infants by parents and caregivers^(30)^.

### Study limitations

Given the current configuration of the application, one limitation was a bias in the validation process by information technology experts, as the application was still in website format and was made available to the experts without password protection or access keys to the technology’s database. As a result, the item “access” received a low score.

Additionally, difficulties in recruiting and receiving timely feedback from the experts may have interfered with the evaluation and improvement of the technology, considering that all research requires time to be completed and refined. Thus, no feedback was received on the adjusted version of the technology following the expert group review. Another limitation was the low number of experts who agreed to participate in the study, despite a large number having been invited.

### Contributions to the Field, Health, or Public Policy

The BASENP app is a tool that can contribute to the knowledge and care provided by parents and caregivers of infants, offering reliable and accessible information. Therefore, its future availability on app store platforms across different operating systems may support the monitoring of child development and promote healthy growth and development.

## CONCLUSIONS

The BASENP application was developed and validated with methodological rigor, following its five development phases (communication, planning, modeling, construction, and delivery), and was supported by experts in both the health (nursing) and computing fields.

It is believed that the use of the application may contribute to improving the knowledge and care provided to infants by helping parents and caregivers better understand and facilitate the developmental process. It is hoped that this study will serve as a strong incentive for the increased production of research in this thematic area, thus promoting the integration of health technologies aimed at supporting the sensitive periods of child development.

## Data Availability

The research data are available within the article.
