# Supplementary material for: Sensitive periods of child development: a study on the construction and validation of the BASENP mobile application
Source: Rev Bras Enferm. 2025 Dec 8;78(Suppl 4):e20240475. doi: 10.1590/0034-7167-2024-0475 (PMC12700571; doi:10.1590/0034-7167-2024-0475)
Supplement: Translation

# Translation: Períodos sensíveis do desenvolvimento infantil: estudo de construção e validação do aplicativo móvel *BASENP*

**DOI:** 10.1590/0034-7167-2024-0475pt

**Authors:** Carolaine da Silva Souza, Paulo Roberto Gomes Abreu, Edmara Chaves Costa, Hévila Ferreira Gomes Medeiros Braga, Francisco Arnoldo Nunes de Miranda, Antônio Carlos da Silva Barros, Flávia Paula Magalhães Monteiro

**Affiliations:** I Universidade Federal do Rio Grande do Norte. Natal, Rio Grande do Norte, Brasil; II Universidade da Integração Internacional da Lusofonia Afro-Brasileira. Redenção, Ceará, Brasil

**Keywords:** Desenvolvimento Infantil, Estudo Metodológico, Tecnologia da Informação, Aplicativos Móveis, Enfermagem.

## Abstract

**Objetivos:**

descrever a construção e validação do aplicativo móvel sobre períodos sensíveis do desenvolvimento infantil.

**Métodos:**

pesquisa metodológica de produção tecnológica e validação de um *software*, do tipo aplicativo móvel, no período de março a outubro de 2023, realizada em duas etapas: 1) Construção do aplicativo móvel *BASENP* e 2) Validação de conteúdo e aparência entre os especialistas.

**Resultados:**

os especialistas de conteúdo possibilitaram a avaliação do material com Índice de Validade do Conteúdo total de 0,89; já os especialistas da área de tecnologia da informação e comunicação obtiveram Índice de Concordância de 0,86%, teste binomial não significativo para maioria dos itens, de ambos os grupos.

**Conclusões:**

o aplicativo foi construído e validado com rigor metodológico e poderá contribuir para a melhoria do conhecimento e assistência prestada ao lactente.

## INTRODUÇÃO

O desenvolvimento infantil é compreendido como um processo complexo, contínuo, dinâmico e progressivo, que reúne um conjunto de mudanças inicialmente simples, evoluindo para o alcance progressivo de maiores habilidades. Por não se tratar de um processo linear e uniforme, passa por fases de ruptura, mutação e adaptação resultantes da dinâmica relacional de diversos fatores fisiológicos na vida da criança^(1-3)^.

Dessa forma, ao alcançar uma nova habilidade (marcos) no desenvolvimento, a criança pode perpassar por um processo de aceleração, evidenciado pela desorganização no comportamento, que se projeta como rupturas no desenvolvimento infantil e, a seguir, se sucedem por períodos de reorganização. No geral, são acompanhados por ansiedade e crise parental^(4)^.

Nesse contexto, de um lado, essas habilidades podem ser popularmente conhecidas pelas mães e cuidadores como “saltos no desenvolvimento infantil”, que são períodos sensíveis nos quais a criança ganha novas habilidades cognitivas, motoras, sensoriais, sociais e linguísticas, como aprender novas palavras, escrever, praticar um esporte, dentre outras. Na verdade, são mudanças fisiológicas que sinalizam que a criança está se desenvolvendo de um jeito saudável^(5)^.

Por outro lado, o termo “períodos sensíveis” do desenvolvimento infantil ainda é obscuro e desconhecido pelas mães/cuidadores. Dessa forma, faz-se necessária a difusão de educação em saúde entre pais e cuidadores para haver a implantação e o conhecimento desse termo, para esclarecer que tais termos se referem a novas habilidades que o bebê está adquirindo em cada fase^(6)^.

Desse modo, os pais e cuidadores precisam entender todas as dimensões do desenvolvimento apresentadas em uma criança, que seguem uma sequência definida e previsível, ou seja, elas rastejam antes de engatinhar, engatinham antes de ficar de pé e ficam de pé antes de andar^(7,8)^.

À medida que a criança perpassa por esses momentos fisiológicos, apresenta diferentes comportamentos, tais como: modifica abruptamente seu padrão de sono; chora com mais frequência; fica mais irritada sem motivo aparente, entre outros. No geral, apresentam características de necessidade de apego, atenção e afeto^(9)^. Nesse sentido, os pais tornam-se confusos, angustiados e, equivocadamente, não conseguem distinguir qual fase de desenvolvimento o bebê está passando, considerando esse processo como “patológico”^(10)^.

Assim, os pais, na maioria das vezes, recorrem aos meios de comunicação informais, como as redes sociais e sites de blogs pessoais, deparando-se com informações não confiáveis, que podem gerar mais medo e dúvidas, ao invés de tranquilizar com informações precisas e científicas^(5)^.

Por outro lado, observa-se ainda escassez de evidências científicas sobre os períodos sensíveis acessíveis a esse público, restringindo-se a conteúdos popularizados em conversas pontuais de grupos de mães que vivenciaram o mesmo problema. E, somando-se a isso, o tema é pouco explorado durante consultas ao profissional de saúde e atividades de educação em saúde, nos serviços de atendimento à criança^(6)^.

Desse modo, o impacto das tecnologias na saúde das crianças é um tema de crescente importância, principalmente em uma era onde dispositivos móveis dominam o cotidiano da população. Em síntese, pode-se entender que o uso destas ferramentas propicia grande apoio e auxílio aos pais e cuidadores, pois a utilização de recursos tecnológicos no processo de ensino e aprendizagem é cada vez mais necessária^(11)^.

Assim, o presente estudo se justifica pela necessidade de fomentar conhecimento e produzir uma tecnologia, como o aplicativo móvel para pais/cuidadores das crianças sobre os períodos sensíveis do desenvolvimento infantil, levando-se em conta as dificuldades encontradas na assistência ao lactente, visto que é uma temática ainda incipiente nas rodas de conversa no contexto familiar.

## OBJETIVOS

Descrever a construção e validação do aplicativo móvel sobre períodos sensíveis do desenvolvimento infantil.

## MÉTODOS

### Aspectos éticos

O estudo foi conduzido segundo as diretrizes de ética nacionais e internacionais e aprovado pelo Comitê de Ética em Pesquisa (CEP) da Universidade da Integração Internacional da Lusofonia Afro-Brasileira (UNILAB), cujo parecer está anexado à presente submissão, e seguiu a Resolução nº 466/12. Todos os participantes assinaram o Termo de Consentimento Livre e Esclarecido (TCLE) antes de sua inclusão no estudo.

### Desenho, local do estudo e período

Pesquisa metodológica de produção tecnológica e validação de um software, do tipo aplicativo móvel^(12)^, realizada em duas etapas: 1) Construção do aplicativo móvel BASENP, que ocorreu entre março e outubro de 2023; e 2) Validação de conteúdo e aparência entre os especialistas, realizada no período de setembro a outubro de 2023, de forma online. O instrumento do Equator TRIPOD foi utilizado para guiar a metodologia, visto que essa ferramenta fornece orientação para a construção de estudos que desenvolvem, validam ou atualizam um modelo de predição multivariável^(13)^.

### População ou amostra; critérios de inclusão e exclusão

Após construído, o aplicativo móvel foi validado por um comitê composto por especialistas da área da saúde, sendo enfermeiros que possuíam experiência clínica e conhecimento teórico sobre desenvolvimento infantil de, no mínimo, um ano, tese/dissertação na área do desenvolvimento infantil e publicações nessa temática, e por especialistas da tecnologia da informação, com ênfase na produção, avaliação e/ou implementação de software, conforme os critérios de Fehring^(14)^.

A seleção dos especialistas para validação seguiu as recomendações da NBR ISO/IEC 14598-6, que preconiza um mínimo de oito membros para cada grupo de avaliadores, a fim de haver representatividade da categoria de usuários de *software* ^(15)^.

### Protocolo do estudo

Este estudo seguiu as cinco etapas propostas por Pressman e Maxim: comunicação, planejamento, modelagem, construção e entrega^(12)^. Na primeira etapa, foi realizada uma busca nas lojas virtuais de aplicativos: *Apple Store* e *Play Store*, para verificar a existência de aplicativos com temática semelhante. Porém, foi encontrado apenas um, que era pago, em outro idioma e construído apenas para fins comerciais.

Ressalta-se que, antes de confirmar o nome do aplicativo, foi realizada uma busca nessas mesmas plataformas para verificar se já havia alguma patente ou material infantil com esse nome.

Assim, realizou-se a construção da revisão de escopo para compor o conteúdo da tecnologia, conforme registro na plataforma OSF, por meio do protocolo: https://doi.org/10.17605/OSF.IO/3JTDZ. A partir dessa revisão, definiu-se como conteúdo do aplicativo os conceitos sobre os períodos sensíveis, quantidade de períodos, tempo de ocorrência, os domínios do desenvolvimento infantil, marcos do desenvolvimento infantil, reações do bebê em cada fase, orientações para os pais lidarem com os períodos sensíveis e a importância da participação da família nesse processo.

Complementarmente, foi realizado também um levantamento prévio do conhecimento dos pais e cuidadores, com o intuito de identificar suas principais necessidades sobre a temática, para compor a estrutura conceitual da tecnologia. Assim, tais resultados encontram-se descritos no estudo mais amplo, fruto da dissertação de mestrado^(16)^.

O aplicativo foi construído em parceria com o curso de Engenharia da Computação da Universidade da Integração Internacional da Lusofonia Afro-Brasileira (UNILAB), com ajuda de um docente do referido curso e um discente voluntário. Utilizando a ferramenta Figma, uma plataforma online para criação de interfaces de protótipos, foi necessário construir um modelo ou protótipo do que se desejava, incluindo as funções e atividades que seriam desempenhadas pelo *software*.

Além disso, foi utilizado, como modelo de processo, o modelo incremental, que auxilia no rápido fornecimento de um determinado conjunto funcional aos usuários, para só depois refinar e expandir suas funções em versões subsequentes^(17)^.

Na etapa de validação da tecnologia, para o recrutamento dos profissionais especialistas, utilizou-se a seleção por meio da Plataforma Lattes, na qual os primeiros participantes foram escolhidos por meio das palavras-chave “saúde da criança”, “construção”, “desenvolvimento infantil” e “tecnologia de informação e comunicação”, que foram as primeiras “sementes” do estudo. Para complementar, utilizou-se também a técnica “bola de neve”, em que os profissionais participantes desta etapa indicam outros com o mesmo perfil para contribuírem na apreciação do material^(18)^.

Os especialistas selecionados receberam uma carta-convite - 50 cartas para cada grupo de especialistas, totalizando 100 convites -, o TCLE e instruções sobre o estudo, via correio eletrônico. Posteriormente, após o retorno do TCLE assinado, foram disponibilizados o instrumento de avaliação e o link para acesso ao aplicativo. Facultou-se um prazo de 30 dias para o retorno do material apreciado, conforme os objetivos pretendidos.

A validação do aplicativo pelos especialistas foi dividida em três partes: 1) Levantamento do perfil do profissional (ocupação atual, área de atuação, titulação, tempo de formação, tipo de experiência); 2) Avaliação de conteúdo por meio de instrumento adaptado e utilizado em outros estudos de validação de aplicativos móveis^(19,20)^, composto por 18 itens divididos nos seguintes domínios: objetivos, estrutura, apresentação e relevância; 3) Apreciação do aplicativo quanto aos aspectos: funcionalidade, confiabilidade, usabilidade, eficiência, manutenibilidade e portabilidade do aplicativo^(14)^.

Por fim, cada item do instrumento de avaliação supracitado foi pontuado numa escala Likert de cinco pontos, conforme apreciação dos especialistas, variando entre “nem um pouco apropriado” até “completamente apropriado”^(21)^.

### Análise dos resultados e estatística

O banco de dados foi organizado no Microsoft Excel e a análise dos dados foi feita no *Statistical Package for the Social Sciences* (SPSS), versão 28.0. Foi calculado o Índice de Validade de Conteúdo (IVC) para estimar o grau de concordância dos itens pelos especialistas, sendo considerado válido o item com valor igual ou superior a 0,80^(19)^. Além disso, empregou-se o teste exato de distribuição binomial indicado para pequenas amostras, estatisticamente significante com p>0,05 e referência de 0,75 de concordância para estimar a confiabilidade estatística dos IVC^(22)^.

## RESULTADOS

Conforme o processo de construção e validação do aplicativo móvel, a construção do conteúdo/estrutura conceitual do *software* foi realizada mediante 30 estudos científicos e outros materiais, como livro-texto e sites, e se deu por meio das seguintes etapas:

### Comunicação

Definiu-se a estrutura das informações da tecnologia no que se refere ao tipo de linguagem, e estabeleceu-se que o aplicativo seria voltado para pais e cuidadores de crianças na fase lactente. Além disso, foram definidos os requisitos que o aplicativo proposto teria, sendo eles: interface para cadastro; registros de informações adicionais; informações sobre os períodos sensíveis; materiais complementares; termos e políticas de privacidade.

### Planejamento

O planejamento ocorreu sobre a estrutura de *layout*, incluindo cores e imagens, seguindo um roteiro sobre o conteúdo previamente elaborado. O material informativo foi baseado na revisão de escopo, descrita na primeira etapa desta pesquisa.

### Modelagem

Nessa etapa, foi realizada a prototipação do aplicativo móvel, com a escolha de interfaces inseridas no aplicativo. Para tanto, foi necessário o auxílio de uma profissional *web designer* na construção dos modelos previamente definidos na etapa de planejamento e para as imagens.

Ao todo, foram 17 interfaces na composição inicial do protótipo, com paletas de cores rosa neutro e rosa bebê, e ícones de bebê, usuário, sino, configurações, registros e calendário. As ilustrações do aplicativo foram escolhidas para promover diversidade e inclusão social do público.

### Construção

Nessa etapa, os autores envolvidos na construção da tecnologia utilizaram a metodologia *SCRUM*, um método ágil, utilizado em projetos mais complexos, com a definição dos conceitos básicos. Para isso, foram realizadas as seguintes atividades principais: interfaces simples e intuitivas, construção para vários ecossistemas, utilização de ferramenta moderna e escolha desta ferramenta chamada *Flutter* ^(23)^.

Na interface inicial, localiza-se o menu com todas as informações, criado para ressaltar as imagens da interface inicial e para melhor entendimento dos usuários. Em seguida, constam os períodos sensíveis, contendo todas as informações pertinentes sobre a temática. Então, qualquer dúvida que os pais/cuidadores tiverem, irão navegar por essa interface e escolher qual opção selecionar.

Destarte, na outra interface, *Domínios do desenvolvimento infantil*, caracteriza-se cada um destes: o físico, cognitivo, socioemocional e psicossocial; na seguinte interface, contém os marcos do desenvolvimento infantil desde o nascimento até os dois anos. Nesta tela, existem orientações aos pais sobre como ajudar seu filho no momento de uma reação de um período sensível e, por último, a interface contendo a importância dos cuidadores nesse processo de desenvolvimento de seus filhos(as).

Prosseguindo, encontra-se a interface para os pais registrarem as informações que preferirem sobre quais comportamentos o bebê está apresentando ou alguma dúvida que surja, contendo: o perfil do usuário, onde ele irá se registrar e colocar suas informações. Constam, também, os termos de uso do usuário e garantia do sigilo, como política de privacidade.

Quanto à paleta de cores, foi pensado em cores verdes, *sarje* e uma cor neutra, que pudesse ser utilizada para ambas as crianças, sem distinção de sexo, o corpo textual do roteiro, que compõe as informações do aplicativo de forma atrativa, e imagens amplamente difundidas do pai, mãe, avô, assim como a faixa etária da criança, raça, diversidade cultural, inclusão social e cor.

A posteriori, os próprios autores definiram o nome para o aplicativo, nomeado *BASENP APP*, para refletir o conteúdo retratado. Assim, a sigla “BA” refere-se a *babys* (bebê), “SEN” a *sensitive* (sensíveis) e “P” a *periods* (períodos), resultando em “períodos sensíveis do bebê” (*Babys sensitive periods*).

### Entrega

Na etapa de entrega, o aplicativo está configurado em formato de site (http://periodossensiveis.infinityfreeapp.com/#/config/), podendo ser acessado por todo tipo de aparelho desde computadores, até mesmo pelos telefones móveis, conforme demonstrado na Figura 1.

**Figura 1:** Aplicativo móvel BASENP

### Validação de conteúdo e aparência do aplicativo móvel entre especialistas

Foram convidados 100 especialistas; entretanto, apenas 17 aceitaram participar da apreciação do aplicativo móvel, sendo nove enfermeiros com expertise na saúde da criança e desenvolvimento infantil, e oito profissionais da área da computação, alcançando o número mínimo definido para cada grupo.

Entre os enfermeiros - dos quais oito eram do sexo feminino e um do sexo masculino - a idade variou entre 24 e 58 anos, com média de 36,11 anos. Em relação ao tempo de obtenção do título, houve variação de 2007 a 2022. Quanto à titulação acadêmica dos especialistas da área da saúde, quatro eram mestres, três possuíam doutorado e dois tinham pós-doutorado.

Ressalta-se ainda que, entre os especialistas da área da saúde, cinco também eram docentes, sendo que três deles atuavam no ensino e na pesquisa.

A seguir, a Tabela 1 apresenta a avaliação de concordância dos especialistas acerca do conteúdo do aplicativo móvel.

**Tabela 1:** Validação das características dos itens, Índice de Validade de Conteúdo pelos especialistas de conteúdo, Redenção, Ceará, Brasil, 2023

Item	N° de concordância	IVC^*^	Valor de *p* ^**^
**OBJETIVOS**			
1.Os objetivos contemplam o tema proposto	9	1,00	0,075
2.O aplicativo móvel é adequado ao processo conhecimento sobre períodos sensíveis do desenvolvimento	9	1,00	0,075
3.O aplicativo esclarece dúvidas sobre o tema proposto	8	0,89	0,300
4.O aplicativo proporciona reflexão sobre o tema	9	1,00	0,075
5.O aplicativo contribui para o conhecimento de pais e/ou cuidadores	9	1,00	0,075
**ESTRUTURA/APRESENTAÇÃO**			
6.A linguagem é adequada ao público-alvo	6	0,67	0,010
7.A linguagem é adequada ao material abordado	8	0,89	0,300
8.A linguagem é interativa, permitindo envolvimento ativo no processo ensino aprendizagem	7	0,78	0,601
9.O aplicativo possui informações corretas	8	0,89	0,300
10.O aplicativo possui informações objetivas	8	0,89	0,300
11.O aplicativo possui informações esclarecedoras	8	0,89	0,300
12.O aplicativo possui informações necessárias	9	1,00	0,075
13.O aplicativo apresenta uma sequência lógica de ideias	9	1,00	0,075
14.O tema é atual	9	1,00	0,075
15.A quantidade de texto é adequada	7	0,78	0,601
**RELEVÂNCIA**			
16.O aplicativo estimula o entendimento do tema	9	1,00	0,075
17.O aplicativo contribui para o conhecimento na área	9	1,00	0,075
18.O aplicativo desperta o interesse pelo tema	9	1,00	0,075
**PARTE III -FUNCIONALIDADE**			
19.O aplicativo é fácil de ser manuseado	8	0,89	0,300
20.O aplicativo é preciso na execução de suas funções	8	0,89	0,300
21.O aplicativo apresenta as principais funções necessárias para o processo ensino-aprendizagem sobre o tema	8	0,89	0,300
**CONFIABILIDADE**			
22.O aplicativo reage adequadamente quando ocorrem falhas	8	0,89	0,300
23.O aplicativo informa ao usuário a entrada de dados inválidos	8	0,89	0,300
**USUABILIDADE**			
24.É fácil de aprender a usar o aplicativo	8	0,89	0,300
25.O aplicativo oferece ajuda de forma clara	6	0,67	0,010
**EFICIÊNCIA**			
26.O tempo de execução do aplicativo é adequado	9	1,00	0,075
27.Os recursos disponibilizados no aplicativo são adequados	7	0,78	0,601
**IVC TOTAL**		**0,89**	

* Índice de Validade de Conteúdo;

** Teste binomial.

Mediante o exposto, é possível observar que os itens 6 (IVC = 0,67), 8 (IVC = 0,78), 15 (IVC = 0,78) e 25 (IVC = 0,67) não obtiveram concordância pelos especialistas, no que se refere à “linguagem adequada ao público-alvo”, “linguagem interativa, permitindo envolvimento ativo no processo ensino-aprendizagem”, “quantidade de texto adequada” e se “oferece ajuda de forma clara”, não atingindo o escore maior que 0,80, como adotado previamente neste estudo. No teste binomial, observou-se discordância significativa no item 6 (p = 0,010).

Assim, pode-se considerar que os demais itens avaliados foram adequados conforme o índice adotado, pois alcançaram média de escores no IVC total de 0,89, considerado significante quanto à validação de conteúdo e aparência.

Nesta apreciação, os especialistas da área da saúde tiveram um espaço para fazer sugestões, caso achassem pertinente, para a melhoria do aplicativo móvel.

A seguir, na Tabela 2, tem-se a validação de aparência dos especialistas técnicos.

**Tabela 2:** Validação das características dos itens, Índice de Validade de Conteúdo pelos especialistas de aparência, Redenção, Ceará, Brasil, 2023

Item	N° de concordância	IVC^*^	Valor de *p* ^**^
**FUNCIONALIDADE**			
1.O aplicativo é preciso na execução de suas funções	8	1,00	0,100
2.O aplicativo executa de forma correta o que foi proposto	7	0,88	0,267
3.O aplicativo dispõe de segurança de acesso por meio de senhas	3	0,38	0,023
**CONFIABILIDADE**			
4.O aplicativo reage adequadamente quando ocorrem falhas	7	0,88	0,267
5.O aplicativo informa ao usuário a entrada de dados inválidos	6	0,75	0,311
**USABILIDADE**			
6.É fácil entender o conceito de períodos sensíveis do desenvolvimento infantil e aplicação do aplicativo	8	1,00	0,100
7.É fácil aprender a usar o aplicativo	8	1,00	0,100
8.O aplicativo oferece ajuda de forma clara	7	0,88	0,267
9.É fácil de operar e controlar o aplicativo	8	1,00	0,100
**EFICIÊNCIA**			
10.O tempo de execução do aplicativo é adequado	7	0,88	0,267
11.Os recursos disponibilizados são adequados	8	1,00	0,100
**MANUTENIBILIDADE**			
12.É fácil encontrar uma falha quando ocorre	7	0,88	0,267
13.É fácil modificar e adequar o aplicativo quando Necessário	8	1,00	0,100
14.É fácil testar quando há alterações no aplicativo	8	1,00	0,100
**PORTABILIDADE**			
15.É fácil adaptar o aplicativo a outros ambientes	7	0,88	0,267
16.É fácil instalar o aplicativo em outros dispositivos	7	0,88	0,267
**IVC TOTAL**		**0,86**	

* Índice de Validade de Conteúdo;

** Teste binomial.

A partir disso, é possível observar que os itens 3 (IVC = 0,38) e 5 (IVC = 0,75) não obtiveram concordância pelos especialistas. No teste binomial, observou-se discordância significativa no item 3 (p = 0,027).

Assim, pode-se considerar que os demais itens avaliados foram adequados conforme o índice adotado, pois alcançaram média de escores no IVC total de 0,86, considerado significante quanto à validação técnica do aplicativo.

Quanto às sugestões de melhorias do aplicativo feitas pelos especialistas, destacam-se: revisão ortográfica; associação de imagens à faixa etária da criança; inclusão de links, materiais complementares, vídeos, avatares das crianças e permissão para alteração da data dos registros. Essas foram as principais sugestões acatadas pelos autores, sendo realizada a retroalimentação do aplicativo a partir das contribuições dos especialistas.

## DISCUSSÃO

O aplicativo móvel é um programa computacional desenvolvido para ser utilizado em dispositivos móveis, como smartphones, tablets, computadores, dentre outros meios, que possui recursos projetados com a finalidade de serem usados em contextos de ensino-aprendizagem. Assim, o BASENP foi desenvolvido com o intuito de fornecer educação em saúde aos usuários, na tentativa de preencher a lacuna ainda existente na literatura^(24)^.

Quanto ao desenvolvimento do protótipo do aplicativo, foi pensado em um layout que chamasse a atenção dos usuários e trouxesse interatividade, sendo inclusos vídeos do Ministério da Saúde, imagens que retratam a realidade das famílias, bem como a inclusão das populações vulneráveis, com imagens de crianças negras, com deficiência, albinas, dentre outras. Estudos que construíram aplicativos voltados para a saúde da criança ressaltam a importância de materiais complementares para dinamizar a ferramenta^(25,26)^.

Estudo indicou que abordar o design e o conteúdo do aplicativo facilita a acessibilidade e a boa navegação através das telas, considerando que o protótipo fará parte de um aplicativo voltado para pais e/ou cuidadores e necessita ter uma interface interativa e fácil de ser utilizada, especialmente quando se leva em consideração o fato de muitos pais não estarem acostumados a experiências com aplicativos móveis^(27)^.

Assim, o conteúdo disposto no aplicativo foi elaborado por meio de uma revisão de escopo, para levantar as fragilidades e potencialidades sobre a temática, contendo desde o contexto dos períodos sensíveis até as reações de cada bebê. Essa mesma estratégia foi utilizada em outro estudo ao construir um aplicativo móvel para avaliação dos pés de pessoas com diabetes mellitus, sendo uma das etapas primordiais para a construção de qualquer tecnologia, usada para nortear o desenvolvimento do aplicativo^(28)^.

Neste contexto, o conhecimento familiar sobre o desenvolvimento infantil, especificamente sobre os comportamentos que os filhos apresentam ao longo de suas etapas, tem fundamental importância, pois influencia as crenças e práticas parentais e, consequentemente, a evolução dos seus filhos^(25)^.

Ademais, enfatiza-se que a construção e a validação de tecnologias são processos essenciais e complexos, que requerem atitudes pedagógicas e método apropriado. Caso não seja realizada a validação, o material torna-se inadequado e não cumpre o objetivo proposto, que é fornecer informações educacionais^(29)^.

Alguns autores citam que a adequação funcional e a confiabilidade de um aplicativo estão diretamente ligadas à possibilidade de a tecnologia prover funções que atendam às necessidades da população. Nesse sentido, é essencial a avaliação da qualidade para averiguar se a tecnologia apresenta funcionalidade adequada de uma ou mais ferramentas^(24)^.

O aplicativo BASENP foi considerado válido, visando contribuir de maneira educacional aos pais/cuidadores no acompanhamento do desenvolvimento dos seus filhos(as), uma vez que fornece informações para solucionar dúvidas que surgirem. Nesse contexto, ressalta-se a importância desta tecnologia no apoio, conhecimento e propagação de informações ao público-alvo^(22)^.

O uso de aplicativo móvel tem potencial de emprego nas atividades de ensino, contribuindo assim para o processo de ensino-aprendizagem e promovendo ainda o desejo pela pesquisa e leitura de informações bibliográficas^(24)^. O desenvolvimento do aplicativo atende à justificativa e à necessidade de fomentar conhecimento e trazer uma tecnologia, como o aplicativo móvel, para pais/cuidadores das crianças sobre a temática em questão.

Sobre a temática objeto do presente estudo, a literatura revela uma lacuna quanto ao desenvolvimento de materiais educativos, bem como de informações sobre o tema, que abordem o cuidado e o acompanhamento pelos pais/cuidadores dos lactentes^(30)^.

### Limitações do estudo

Em face da configuração criada para o aplicativo, houve como limitação, um viés na validação pelos especialistas da tecnologia da informação, pois o aplicativo ainda se encontra em formato de site, disponibilizado para os especialistas o que impedia o uso de senha e chave de acesso ao banco de dados da tecnologia. Dessa forma, o item “acesso” obteve pouca pontuação.

Além disso, a dificuldade de recrutamento e retorno dos especialistas em tempo hábil pode ter interferido na avaliação e aprimoramento da tecnologia, tendo em vista que toda pesquisa exige tempo para ser cumprida e aperfeiçoada. Assim, não houve retorno da tecnologia ajustada após apreciação dos grupos de especialistas. Como também, a baixa quantidade de especialistas que aceitaram participar do estudo, visto que houve o convite de um grande número de especialistas.

### Contribuições para a Área, Saúde ou Política Pública

O *BASENP* é uma ferramenta que pode contribuir com o conhecimento aos cuidados prestados pelos pais/cuidadores aos lactentes, concomitantemente com informações fidedignas e acessíveis. Dessa forma, sua disponibilização futuramente nas plataformas de lojas de aplicativos em diferentes sistemas operacionais, contribuirá com o acompanhamento do desenvolvimento infantil destas crianças e promoção do crescimento e desenvolvimento infantil.

## CONCLUSÕES

O aplicativo BASENP foi construído e validado com rigor metodológico, compreendido nas suas cinco fases de construção (comunicação, planejamento, modelagem, construção e entrega), proporcionado por profissionais especialistas na área da saúde (enfermeiros) e profissionais da área da computação.

Acredita-se que o uso do aplicativo, poderá contribuir para a melhoria do conhecimento e assistência prestada ao lactente, no sentido de facilitar, entender e dinamizar o processo de cuidado por parte dos pais e cuidadores no desenvolvimento da criança. Espera-se que este estudo seja um grande estímulo para uma maior produção de trabalhos nessa área temática, promovendo assim, a imersão das tecnologias em saúde para propiciar o uso de ferramentas voltadas aos períodos sensíveis do desenvolvimento.

## Data Availability

Os dados da pesquisa estão disponíveis no corpo do artigo.
